# 2367. Six-month Humoral Response Following Two Or Three Doses Of Covid-19 Vaccines As Primary Vaccination Followed Or Not By a Booster Dose In Specific Populations – ANRS0001S COV-POPART Cohort Study

**DOI:** 10.1093/ofid/ofad500.1988

**Published:** 2023-11-27

**Authors:** Paul LOUBET, Camille Gilbert, David Laplaud, Martine Laville, Bruno Laviolle, Jean-Daniel Lelièvre, Jean-Yves Blay, Benoit Barrou, Benjamin Terrier, Stéphanie Nguyen Quoc, Aude Barquin, Cécile Moins, Eric Tartour, Xavier de Lamballerie, Odile Launay, L I nda Wittkop

**Affiliations:** CHU de Nîmes, Nimes, Languedoc-Roussillon, France; Université de Bordeaux, INSERM UMS 54 MART, ISPED, Bordeaux, France, Bordeaux, Aquitaine, France; CHU de Nantes, Nantes, Pays de la Loire, France; CHU Lyon, Lyon, Rhone-Alpes, France; CHU Rennes, Rennes, Bretagne, France; CHU Henri Mondor, Creteil, Ile-de-France, France; Unicancer Lyon, Lyon, Rhone-Alpes, France; APHP, Paris, Ile-de-France, France; APHP, Paris, Ile-de-France, France; Hopital Pitié-Salpétrière, Paris, Ile-de-France, France; Université de Bordeaux, INSERM UMS 54 MART, ISPED, Bordeaux, France, Bordeaux, Aquitaine, France; ANRS, Paris, Ile-de-France, France; Service d’Immunologie Biologique, APHP, Hôpital Européen Georges Pompidou, 75015 Paris, France; PARCC, INSERM U970, Université de Paris, 75006 Paris, France, Paris, Ile-de-France, France; APHM, Marseille, Provence-Alpes-Cote d'Azur, France; Université Paris Cité; Inserm F-CRIN, I-REIVAC ; Assistance Publique Hôpitaux de Paris, Paris, Ile-de-France, France; Université Bordeaux, Bordeaux, Aquitaine, France

## Abstract

**Background:**

The duration of humoral response in immunocompromised populations according to the number of doses in the primary vaccine regimen and the number of booster doses is little known.

**Methods:**

Participants from the French national multi-center prospective cohort study ANRS0001S COV-POPART were included (11 specific subpopulations and 2 control groups (18-64 years and over 65 years)). We evaluated the immune response to COVID-19 vaccines up to 6 months after the second dose, distinguishing participants having received two or three doses in their initial vaccination scheme and those with or without a booster dose before 6 months. Participants with positive anti-nucleocapsid (NP) antibodies or SARS-CoV-2 infection during follow-up were excluded. Serological (ELISA EuroImmun®) and seroneutralisation (*in vitro* neutralization assay, original strain) tests were carried out centrally.

**Results:**

3724 participants were included: 2756 from specific subpopulations and 968 controls. Participants in specific sub-populations and the control groups mostly received two doses of BNT162b2 (68.6% and 83.6%, respectively). Fifteen percent of participants in a specific sub-population received 3 doses within their initial vaccine scheme (mostly solid organ transplants (SOT) and Hematopoietic stem cell transplants (HCT)) and 11.2% received a booster dose within 6 months after a median time of 5 months after the last dose. The control groups and all specific sub-populations who received two doses as the primary regimen, except solid SOT and HCT groups, increased their anti-Spike and seroneutralisation titer after a booster dose (Figure 1A and 1C).

The booster dose had little effect on participants who received three doses as a primary vaccination regimen compared to those who did not receive the booster dose (Figure 1B and 1D).

6-month median (IQR) anti-Spike IgG titers (BAU/mL) in specific subpopulations who received two (A) and three doses (B) within their initial vaccine scheme. 6-month seroneutralisation titers in specific subpopulations who received two (C) and three doses (D) within their initial vaccine scheme.

**Figure:**
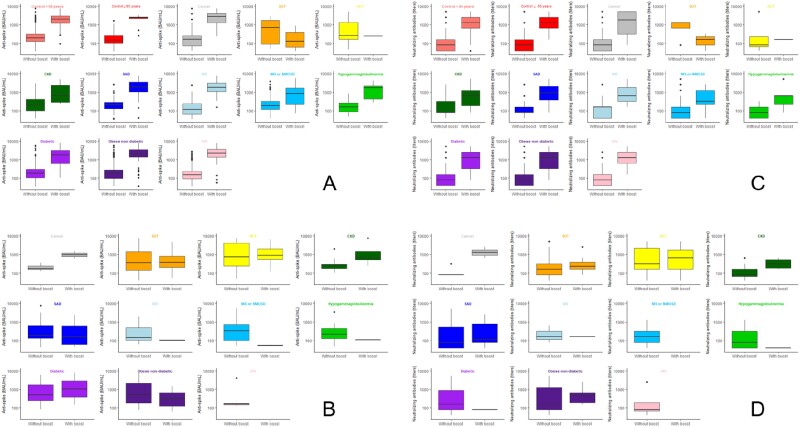

Solid organ transplantation (SOT), hematopoietic stem cell transplantation (HCT), chronic renal failure (CKD), systemic autoimmune diseases (SAD), inflammatory rheumatic diseases (IRD), multiple sclerosis (MS) or neuromyelitis optica spectrum disorders (NMOSD).

**Conclusion:**

A booster dose in those who received 2 doses as a primary vaccination regimen increased 6-month humoral responses in almost all sub-populations except SOT and HSCT. The effect of booster doses in those receiving three doses as a primary vaccination regimen was low, reflecting the profound immunosuppression of these patients.

**Disclosures:**

**Paul LOUBET, MD, PhD**, Astrazeneca: Advisor/Consultant|Astrazeneca: Board Member|Moderna: Board Member|Pfizer: Advisor/Consultant|Pfizer: Board Member **Odile Launay, MD, PhD**, Moderna: Advisor/Consultant|Pfizer: Advisor/Consultant|Pfizer: Board Member

